# Potential Molecular Targets of Oleanolic Acid in Insulin Resistance and Underlying Oxidative Stress: A Systematic Review

**DOI:** 10.3390/antiox11081517

**Published:** 2022-08-03

**Authors:** Ángel Fernández-Aparicio, María Correa-Rodríguez, Jose M. Castellano, Jacqueline Schmidt-RioValle, Javier S. Perona, Emilio González-Jiménez

**Affiliations:** 1 Department of Nursing, Faculty of Health Sciences, Melilla Campus, University of Granada, 52005 Melilla, Spain; anfeapa@ugr.es; 2 Instituto de Investigación Biosanitaria (ibs.GRANADA), 18014 Granada, Spain; macoro@ugr.es (M.C.-R.); emigoji@ugr.es (E.G.-J.); 3 Department of Nursing, Faculty of Health Sciences, University of Granada, 18016 Granada, Spain; jschmidt@ugr.es; 4 Department of Food and Health, Instituto de la Grasa-CSIC, Campus of the University Pablo de Olavide, 41013 Seville, Spain; perona@ig.csic.es

**Keywords:** *Olea europaea*, bioactive compounds, triterpenes, oleanolic acid, insulin resistance, type 2 diabetes mellitus, oxidative stress, inflammation, insulin signaling, pathways

## Abstract

Oleanolic acid (OA) is a natural triterpene widely found in olive leaves that possesses antioxidant, anti-inflammatory, and insulin-sensitizing properties, among others. These OA characteristics could be of special interest in the treatment and prevention of insulin resistance (IR), but greater in-depth knowledge on the pathways involved in these properties is still needed. We aimed to systematically review the effects of OA on the molecular mechanisms and signaling pathways involved in the development of IR and underlying oxidative stress in insulin-resistant animal models or cell lines. The bibliographic search was carried out on PubMed, Web of Science, Scopus, Cochrane, and CINHAL databases between January 2001 and May 2022. The electronic search produced 5034 articles but, after applying the inclusion criteria, 13 animal studies and 3 cell experiments were identified, using SYRCLE’s Risk of Bias for assessing the risk of bias of the animal studies. OA was found to enhance insulin sensitivity and glucose uptake, and was found to suppress the hepatic glucose production, probably by modulating the IRS/PI3K/Akt/FoxO1 signaling pathway and by mitigating oxidative stress through regulating MAPK pathways. Future randomized controlled clinical trials to assess the potential benefit of OA as new therapeutic and preventive strategies for IR are warranted.

## 1. Introduction

Diabetes mellitus (DM) is a major public health problem since it currently affects about 420 million people worldwide, and its prevalence is expected to increase by approximately 38% by 2030 [1]. Around 90% of DM world cases are accounted for by type 2 diabetes mellitus (T2DM) [2]. Insulin resistance (IR) affects individuals for many years before the development of T2DM [3], and consists of a diminished sensitivity of insulin target tissues to healthy insulin levels [4]. IR disrupts the insulin receptor substrate (IRS)/phosphatidylinositol-3-kinase (PI3K)/protein kinase B (Akt) pathway activation [5,6], which provokes a reduction of the glucose uptake in the adipose tissue and skeletal muscle, an increment of the gluconeogenic and glycolytic activity in the liver, and lipid metabolism disturbances in the liver and adipose tissue [7,8].

Authors have proposed possible hypotheses with regard to the development of IR, such as fat accumulation in the liver and skeletal muscle [4]. Obesity-associated chronic inflammation of insulin target tissues implies an impairment of glucose and lipid metabolism, a blockage of insulin signaling, and lower insulin sensitivity [9], since accumulation of fatty acids favors the activation of c-Jun N-terminal kinase (JNK) and of inhibitor of κB kinase (IKK) [8]. Phosphorylation of IKK activates the nuclear transcription factor kappa B (NF-кB) pathway, which upregulates the induction of the typical inflammatory status in IR through releasing proinflammatory cytokines such as interleukin-1β (IL-1β), interleukin-6 (IL-6), and tumor necrosis factor-α (TNF-α) [10]. Moreover, JNK, the extracellular signal-regulated kinase 1/2 (ERK1/2), and p38 pathways comprise the pathway known as mitogen-activated protein kinases (MAPKs) [11], whose impairment has been reported to be involved in inducing IR [10].

The insulin-resistant state is closely linked to the overproduction of reactive oxygen species (ROS) and endoplasmic reticulum (ER) stress in insulin target tissues, which also leads to the activation of JNK and IKK [8]. In this line, elevated levels of proinflammatory cytokines, oxidative stress (OS), and ER stress have been suggested to trigger IR by negatively regulating insulin signaling [12]. In this way, a vicious circle is established by which IR increases the OS, which in turn aggravates the insulin-resistant state [13]. Moreover, research highlighting the relevance of OS in the induction of IR has gained importance in the last few years [13,14,15,16]; in adults, it has been reported that IR is significantly associated with OS parameters [17,18].

Early and appropriate management of IR is essential not only to avoid the development of T2DM, but also to avoid the associated macrovascular and microvascular complications. Dietary and lifestyle interventions have been shown to be effective in the prevention and treatment of T2DM [3,19]. When these interventions fail, pharmacotherapy is considered, whose administering over a prolonged period of time unavoidably leads to the occurrence of undesirable adverse effects. Moreover, it has been suggested that the combination of drugs with bioactive compounds could be more efficient [20]. In the last few years, there has been increasing clinical interest in the potential properties of pentacyclic triterpenes to fight metabolic syndrome [21]. The study conducted by Claro-Cala et al. [22] demonstrated that the application of an olive pomace oil containing triterpenic acids, among them maslinic acid and oleanolic acid (OA), attenuated IR and adipose tissue inflammation in diet-induced obese mice. In this line, a randomized controlled trial known as the PREDIABOLE study reported a 55% lower risk of developing T2DM and an improvement of IR state in 176 prediabetic participants after consuming OA-enriched olive oil for 30 months [23]. 

Several studies have focused on analyzing the antidiabetic [24], anti-obesity [25], anti-inflammatory [24], and antioxidant properties [21,25] of OA, which may exert a positive effect in the treatment and prevention of IR. Interestingly, authors have proposed prophylactic and therapeutic roles of OA and its derivatives in several chronic diseases such as ulcerative colitis, multiple sclerosis, metabolic disorders, diabetes, hepatitis, different cancers, and osteoporosis [26,27,28]. Note that our group has also previously reviewed the potential protective effect of OA on the components of MetS. In this systematic review, we found that OA may improve blood pressure levels, hypertriglyceridemia, hyperglycemia, oxidative stress, and IR [29]. Furthermore, in a recent review, we summarized the biological activities of OA and its underlying mechanisms of action [30]. However, to our knowledge, no previous study has systematically examined the molecular targets of OA in insulin resistance and underlying oxidative stress. Taking into account that IR precedes the development of T2DM, a better understanding of potential molecular targets by which OA exerts its ameliorating actions on insulin resistance and underlying OS is needed. On the other hand, the great variability of the studies that continue to be carried out must be considered; thus, it is essential to study the latest original studies published in depth. Therefore, the aim of this study is to systematically review the effects of OA on the molecular mechanisms and signaling pathways involved in the development of IR and underlying OS in insulin-resistant animal models or cell lines. 

## 2. Materials and Methods

A systematic review was conducted following the Preferred Reporting Items for Systematic Reviews and Meta-analysis (PRISMA) 2020 protocol [31]. The protocol of this systematic review is published in PROSPERO (reference CRD42022344225).

### 2.1. Search Strategy

The bibliographic search was carried out in five electronic databases: PubMed, Web of Science, Scopus, Cochrane Plus, and CINAHL. All publications in English between January 2001 and May 2022 were included. The following categories were used in the database search strategy: oleanolic acid AND (“insulin resistance” OR “oxidative stress” OR “reactive oxygen species” OR cytokines OR antioxidants OR kinases). Table 1 shows information on the additional filters used and on the search field in which the search strategy was applied in each database.

### 2.2. Selection Process—Inclusion and Exclusion Criteria

The selection of potentially relevant studies was conducted in two phases. In the first one, the titles and abstracts were reviewed to assess their eligibility. In case of doubt, the full text was also read. In the second phase, the full text of the records previously selected was analyzed. Á.F.-A. and M.C.-R. independently performed the screening process. A third reviewer (J.M.C.) resolved differences and disagreements by consensus. 

The inclusion criteria were established according to the PICOS framework:

- Patients: insulin-resistant animal models or cell lines.
- Intervention: administration of OA.
- Comparison: control substances and/or another bioactive compound.
- Outcomes: signaling pathways that are altered in states of IR and inflammatory and OS biomarkers (proinflammatory cytokines, antioxidant enzymes, transcription factors, and ROS).
- Study type: experimental studies.

The following exclusion criteria were established:

- Narrative and systematic reviews, letter to editor, book chapter, and other kinds of secondary studies.
- Studies that are focused on the extraction of bioactive compounds from natural plants and on the analysis of their biological activities without a more in-depth explanation of the molecular mechanisms implied.
- Studies that are focused on treating comorbidities or short-term or long-term complications of T2DM, without the approach of analyzing the effects of OA on signaling pathways involved in the development of IR.
- Administration of OA derivatives or OA combined with another bioactive compound.
- Non-English studies.

### 2.3. Data Extraction

Data were extracted once the selection process was finished. Data extracted both from the animal studies and the cell experiments were the following: authors, year of publication, subjects and sample size, type of intervention, period of administration and dosage used, and outcomes obtained (insulin signaling, inflammatory and OS pathways; and inflammatory and OS biomarkers). Two independent reviewers (Á.F.-A. and M.C.-R.) performed the data extraction. A third reviewer (E.G.-J.) resolved differences and disagreements by consensus. A meta-analysis was not possible because of the heterogeneous design of the studies included in the review. Therefore, we carried out a narrative description of each of the results measured.

### 2.4. Study Risk of Bias Assessment

Á.F.-A. and J.S.-R. independently analyzed the risk of bias of all the studies included in this review. A third reviewer (J.S.P.) resolved disagreements by consensus. For this purpose, the SYRCLE’s Risk of Bias (RoB) tool [32] was used for the animal studies. The risk of bias for “in vitro” studies was not assessed because of the absence of a standard risk of bias tool for these kinds of studies. 

SYRCLE’s RoB tool was published by the Systematic Review Centre for Laboratory Animal Experimentation (SYRCLE) [32] for assessing the methodological quality of experimental studies of animals. SYRCLE’s RoB tool contains 10 items, and each of them is rated as a “yes” (low risk of bias), a “no” (high risk of bias), or “unclear” (insufficient information to evaluate risk of bias). These items assess six types of bias: selection bias, performance bias, detection bias, attrition bias, reporting bias, and another bias. 

## 3. Results

### 3.1. Study Selection

**Figure 1:**
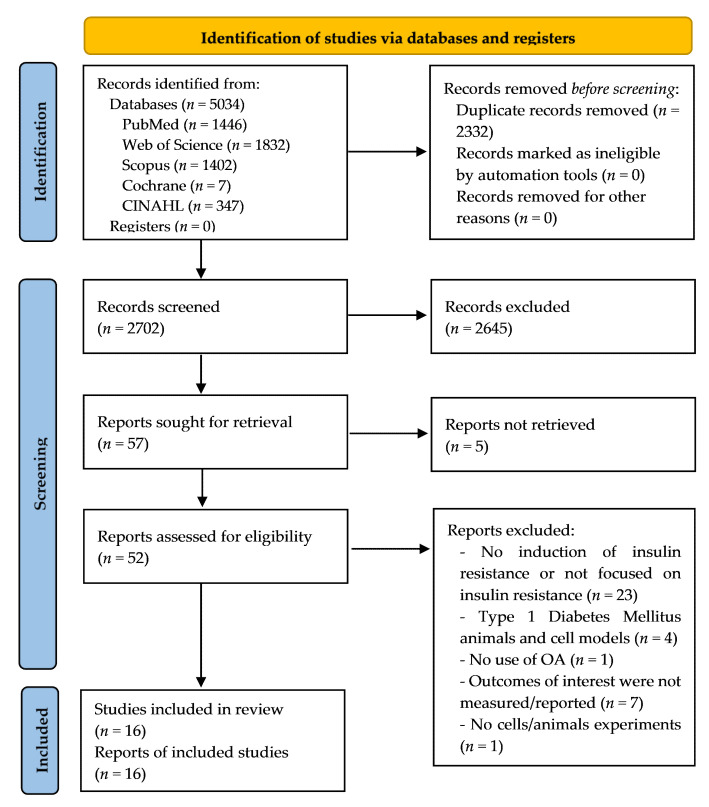
Flow diagram of the study selection process.

Figure 1 shows the flow chart for the process of selection and exclusion of studies according to the PRISMA system [31]. A total of 5034 records were identified: 1446 from PubMed, 1832 from Web of Science, 1402 from Scopus, 347 from CINAHL, and 7 from Cochrane. Once the duplicates were removed, 2702 records were screened, from which 2645 were excluded. Then, 57 reports were sought for retrieval, and 5 of them were not retrieved [33,34,35,36,37]. Later, 52 full-text articles were read to assess their eligibility, and finally 16 studies were included. An inverse search from the included studies was not conducted. 

### 3.2. Characteristics of the Cell Experiments Selected 

The characteristics and the main findings of the cell experiments included in this review are shown in Table 2. Of the three studies selected, two used the HepG2 cell line, and the other one used a human normal hepatocyte line (QZG). To induce IR, different components were used in each study (tert-butyl hydroperoxide (tBHP), sodium oleate, and high concentrations of insulin). 

### 3.3. Characteristics of the Animal Studies Selected

The characteristics of the animal studies selected and their main findings are summarized in Table 3. All of them (*n* = 13) employed rodents, especially rats and mice. Of the 13 studies included, 2 of them employed T2DM mice models. Of the remaining 11, 5 used high-fat diet, 3 used high-fructose diet, 1 used high-fat and fructose diet, 1 used high-fat, high-carbohydrate diet, and 1 used Aroclor 1254 (PCB) to induce IR in rodents. The largest sample size was *n* = 40 and the smallest sample size was *n* = 21. The maximum and the minimum OA dosage administered in the studies were 250 mg/kg/day and 5 mg/kg/day, respectively. The maximum exposition time period to OA was 12 weeks, and it was 1 week for the minimum time period.

### 3.4. Reporting Risk of Bias Assessment 

**Figure 2:**
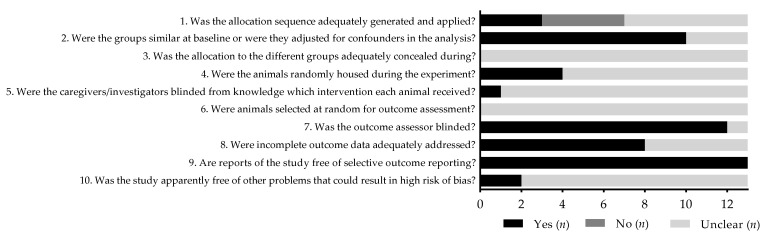
SYRCLE’s RoB tool results for each study. Yes (low risk of bias); No (high risk of bias); Unclear (item not reported, unknown risk of bias); *n* (number of studies).

The results of the risk of bias assessment of the thirteen animal studies included are reported in Figure 2. The SYRCLE’s RoB tool assesses selection bias (items 1–3), performance bias (items 4–5), detection bias (items 6–7), attrition bias (item 8), reporting bias (item 9), and other biases (item 10).

### 3.5. OA Effects in Insulin-Resistant Cell Lines

The IR state was improved by OA actions in the three “in vitro” studies included [38,39,40]. Pretreatment with OA at 10 µM in QZG cells by Wang et al. [38] inhibited the reduction of insulin-stimulated phosphorylation of Akt and ERK induced by tBHP. Li et al. [39], in a study performed on insulin-resistant HepG2 cells, showed an increment in the protein expression of IRS and glucose transporter type 4 (GLUT4) and a decrease of NF-кB protein expression after the administration of OA at 10 µM and 25 µM. Moreover, protein levels of IL-6 and of TNF-α were diminished by OA actions [39]. In another study conducted on insulin-resistant HepG2 cells, OA increased Akt and IRS-1 protein expression and reduced the protein expression of protein tyrosine phosphatase 1B (PTP1B) [40]. 

### 3.6. OA Effects on Impaired Signaling Pathways and on OS in Insulin-Resistant Animal Models

#### 3.6.1. Hepatic IR

Five studies analyzed the actions of OA on livers of insulin-resistant rodents [41,42,43,44,45,46]. The experiments of Wang et al. [41] and Wang et al. [42] reported that OA decreased the protein expression of glucose-6-phosphatase (G-6-Pase) and phosphoenolpyruvate carboxykinase (PEPCK) and increased the AMP-activated protein kinase (AMPK) and Akt phosphorylation in livers of type 2 diabetic mice. In the first one, the following was also reported: a downregulation of the liver gene expression of glycogen phosphorylase (GP), PEPCK1, G-6-Pase, and glucose transporter type 2 (GLUT2), and a non-significant downregulation of peroxisome proliferator-activated receptor γ coactivator 1α (PGC-1α). Moreover, in this study, OA augmented the phosphorylation of acetyl-CoA carboxylase (ACC) and of PI3K, and diminished the phosphorylation of mammalian target of rapamycin (mTOR) and of cAMP-response element-binding protein (CREB) [41]. In the second one, OA also increased the gene expression on mice livers of PGC-1α [42]. 

The administration of OA by Zeng et al. [43] restored the levels of phosphorylated-Akt in livers of type 2 diabetic mice to the same levels as those of non-diabetic mice. These authors also showed an increment of the ratio phosphorylated/total forkhead box O1 (FoxO1), and the diminution of the total FoxO1 protein by OA actions. In the same line, Zhou et al. [44] observed a significant decrease of the hepatic total content of FoxO1, an increase of FoxO1 phosphorylation and acetylation, and a reduction in the gene expression of G-6-Pase during the OA administration and 2 weeks after the OA treatment. They also reported that during the OA administration, the phosphorylation of AMPK-α and of ACC was increased in the livers of type 2 diabetic mice. Similarly, Yunoki et al. [45] evidenced that OA was responsible for downregulating ACC, G-6-Pase, and FoxO1 genes, and for upregulating IRS and AMPK-β-2 genes in the livers of HFD-fed rats.

#### 3.6.2. IR in Adipose Tissue and Skeletal Muscle

Two studies undertook to investigate the IR in adipose tissues [47,48]. Li et al. [47] reported an overexpression of IRS-1, PI3K, and Akt genes after the application of OA for 10 weeks to fructose-induced adipose tissue insulin-resistant rats. In this same study, OA inhibited the phosphorylation of IRS-1 induced by fructose, and increased the ratio of phosphorylated-Akt/Akt. Previous research has shown that OA enhanced the phosphorylation of Akt and reduced the phosphorylation of ERK and JNK in the adipose tissue of HFD-fed mice. Moreover, the HOMA-IR and Adipo-IR index of these mice achieved lower levels because of OA actions [48]. In regards to OA effects on IR in skeletal muscle, Matumba et al. [50] administered OA to high-fructose (HF)-diet-fed neonatal rats, and they observed an increase of the gene expression of AMPK and GLUT4 in the skeletal muscle at the end of the experiment. 

#### 3.6.3. OA Effects on Proinflammatory Cytokines and OS Biomarkers

OS and inflammation in insulin target tissues are closely linked in the establishment of IR. In the study conducted by Wang et al. [42], OA led to a reduction of ROS and L-glutathione oxidized (GSSG), and an increase of glutathione (GSH) in liver mitochondria of treated diabetic mice in comparison to non-OA-treated diabetic mice. OA also increased the protein levels of nuclear factor erythroid 2-related factor 2 (Nrf2), superoxide dismutase (SOD), and catalase (CAT), stabilized those of glutathione cysteine ligase catalytic subunit (GCLC) in the liver of these treated diabetic mice, and decreased serum and liver gene expression of IL-6, IL-1β, and TNF-α [42]. 

Xue et al. [46] demonstrated that the administration of OA for eight weeks mitigated the overexpression of IL-6, TNF-α, and IL-1β in liver tissues of HFD-fed rats. Together with these OA effects, the following were also observed: lower content of malondialdehyde (MDA) and higher levels of SOD, glutathione peroxidase (GPx), and CAT, as well as the inhibition of IкB-α and p65 phosphorylation. Yunoki et al. [45] reported a gene downregulation of TNF-α and IL-1β in livers of HFD-induced rats treated with OA. In the study carried out by Li et al. [48], OA produced a reduction of the gene expression of iNOS, IL-6, TNF-α, IL-1β, and caspase 1 in HFD-fed mice. Moreover, in these mice, OA changed the polarization of adipose tissue macrophages, since macrophages M1 diminished and macrophages M2 increased in these OA-treated HFD-fed mice [48]. 

In the study conducted by Su et al. [49], the exposition to PCB produced in the adipose tissue of mice showed high levels of serum MDA, and also increased the gene expression of NADPH oxidase 4 (NOX4), GCLC, and glutamate-cysteine ligase modifier subunit (GCLM), but it was inhibited by OA. Moreover, it was reported that OA increased the serum SOD and CAT activity in these PCBs-exposed mice. The administration of OA to HF-fed neonatal rats by Nyakudya et al. [51] attenuated the fructose-induced decrease of GSH and CAT activity in the skeletal muscle by the end of the study. In another study performed on HF-fed neonatal rats by Matumba et al. [50], it was observed that OA reduced the plasma levels and gene expression of IL-6 and TNF-α in the skeletal muscle at the end of the experiment.

Gamede et al. [52] induced prediabetic rats through administering high-fat high-carbohydrate (HFHC) diet before the administration of OA. Rats continued to receive either an HFHC diet or a normal diet during OA administration. These authors also observed a mitigation of plasma levels of IL-6, an increase of serum SOD activity, and an increase in GPx activity because of OA actions. In the study performed by Wang et al. [53], OA produced an increase of serum superoxide dismutase (SOD) and CAT activity and a diminution of serum MDA and serum NO in high-fat and fructose (HFF)-diet-fed rats. Moreover, the insulin sensitivity index (ISI) was improved. 

**Figure 3:**
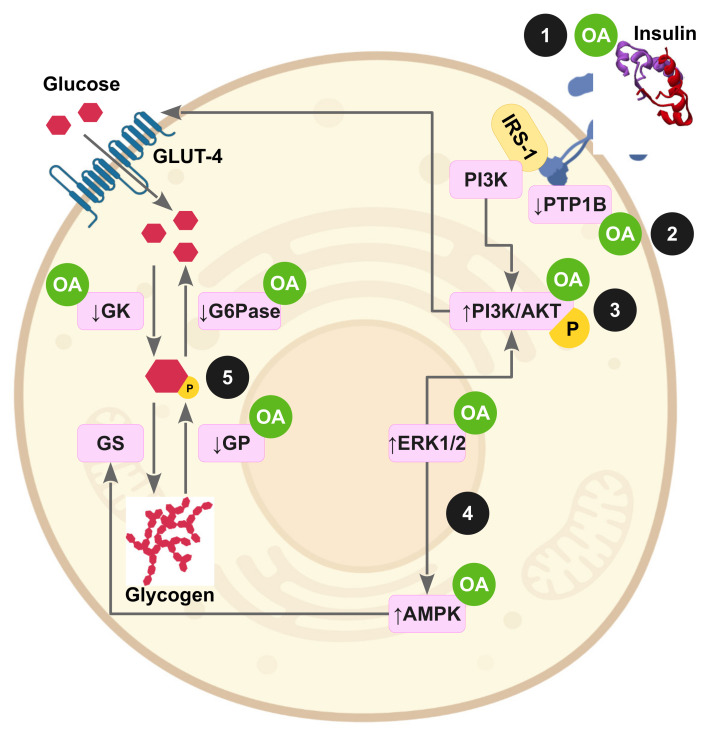
Oleanolic acid improves insulin signaling in peripheral tissues through a multimolecular mechanism. (**1**) OA is an activator of the insulin receptor, exerting an insulin mimetic role; (**2**) OA upregulates insulin sensitivity by inhibition of the tyrosine phosphatase PTP1B and TCPTP; (**3**) OA increases glucose uptake by activation of the PI3K/Akt pathway and GLUT-4 translocation; (**4**) OA also enhances glucose uptake and fatty acid oxidation in muscle and liver by activating the ERK1/2-AMPK axis; (**5**) OA preserves the glycogen pool in muscle and liver by stimulating glucokinase and repressing the glucose-6-phosphatase and glycogen phosphorylase activities.

**Figure 4:**
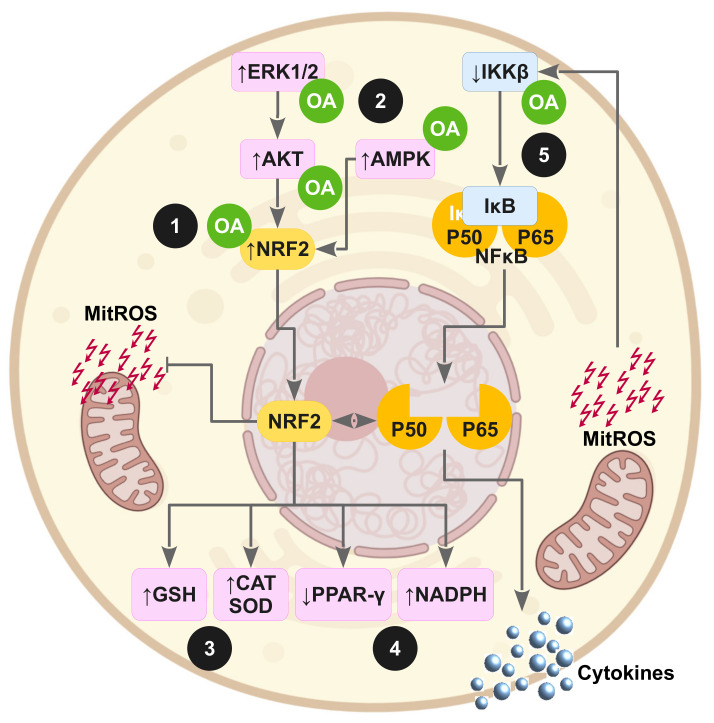
Oleanolic acid exerts antioxidant and anti-inflammatory actions against the supraphysiological production of mitochondrial ROS (mitROS) via transcription factors NRF2 and NFκB. (**1**) OA activates NRF2 by direct interaction with Keap1, the primary sensor that retains NRF2 for ubiquitin-dependent degradation in cytoplasm; (**2**) OA also activates NRF2 through the stimulation of stress kinase pathways such as ERK1/Akt and AMPK; (**3**) OA preserves the glutathione pool and increases the expression of antioxidant enzymes; (**4**) OA conserves the NADPH levels by upregulating genes of the pentose phosphate pathway and downregulating lipogenic genes; (**5**) OA reduces the production of inflammatory mediators, avoiding NFκB activation through competitive inhibition of IKKβ.

Figure 3 and Figure 4 highlight the potential molecular action of OA on impaired insulin signaling pathways and underlying oxidative stress as consequence of the induction of IR in animal experimentation or cell experiments.

## 4. Discussion

This systematic review aimed to provide a better understanding of how OA acts on signaling pathways involved in the development of IR and underlying OS. To our knowledge, no previous studies have been carried out to review the potential molecular targets of OA in IR, as presented in this study. All results provided in this review derive from experimental studies where OA was administered to animal or cell lines induced for IR. The present systematic review shows that OA is capable of attenuating IR through enhancing insulin sensitivity and glucose uptake and suppressing the hepatic glucose production, probably by modulating the IRS/PI3K/Akt/FoxO1 insulin signaling pathway. The modulation of MAPK pathways by OA mitigates underlying OS and inflammation, and consequently improves insulin sensitivity. Therefore, these results suggest the potential molecular targets of the therapeutic and preventive actions of OA on signaling pathways leading to IR. 

In the three “in vitro” studies included, OA attenuated the IR status on hepatic cell lines [38,39,40]. More specifically, the induction of IR to QZG cells by Wang et al. [38] reduced the insulin-stimulated phosphorylation of Akt and ERK, but it was reversed by OA. Another of the studies included was the one conducted by Li et al. [39] on HepG2 cells, where OA attenuated IR through increasing the protein levels of IRS and GLUT4, and repressing the protein expression of NF-кB, IL-6, and TNF-α. It is well known that the activation of the NF-кB pathway negatively affects insulin signaling and promotes the release of proinflammatory cytokines [24,54]. In addition, ROS-induced activation of the NF-кB pathway in skeletal muscle of HFD-fed mice has been associated with increased expression of PTP1B [55], a negative regulator of insulin signaling [56]. In this line, the transfection with PTP1B to HepG2 cells by Niu et al. [57] decreased the transcription of IRS1 and GLUT4. Considering that several authors have previously reported inhibitory activities of OA and its derivatives on PTP1B [56,58,59,60], one of the possible mechanisms by which OA improves insulin sensitivity could be through inhibiting PTP1B. In fact, Zhang et al. [40] showed that OA enhanced the protein levels of IRS and of Akt, and reduced those of PTP1B on IR-induced HepG2 cells. 

In regard to the studies performed on rodents induced for IR, five of them undertook the molecular mechanisms of OA involved in its ability to ameliorate hepatic IR [41,42,43,44,45]. In the studies conducted by Wang et al. [41] and Wang et al. [42], OA led to a decrease of the protein levels of G-6-Pase and PEPCK in type 2 diabetic mice. Moreover, Wang et al. [41] reported that OA had downregulating effects in the liver gene expression of GP, G-6-Pase, GLUT2, and PEPCK1, and reduced mTOR and CREB phosphorylation. The repressive action of OA in the gene expression of G-6-Pase was also observed by Zhou et al. [44] and Yunoki et al. [45]. All these OA-inhibitory effects on gluconeogenic enzymes might be explained by the OA actions on IRS, PI3K, and Akt, since the activation of the insulin IRS-1/PI3K/Akt pathway has been reported to be suppressed in IR [61]. In fact, increased phosphorylation of Akt and of PI3K in livers of type 2 diabetic mice due to OA was reported by Wang et al. [41] and increased phosphorylation of Akt was reported by Wang et al. [42]. The existing literature supports the hypothesis that insulin-stimulated phosphorylation of Akt2 activates glycogen synthase and reduces the transcription of gluconeogenic enzymes through inactivating FoxO1 [62]. Moreover, deacetylation of FoxO1 has been proposed to intensify its activity, favoring gluconeogenesis [63]. Another mechanism by which OA enhances glucose uptake and regulates gluconeogenesis is through increasing phosphorylation of Akt and FoxO1 [43,44], and also acetylation of FoxO1 [44]. Similarly, Yunoki et al. [45] observed gene downregulation of FoxO1 in livers of OA-treated HFD-fed rats. 

Two studies were focused on OA actions in adipose tissue insulin-resistant rodents and they reported an enhancement of the insulin-stimulated phosphorylation of Akt [47,48]. These results are in consonance with those previously discussed for hepatic insulin-resistant rodents [41,42,43,44]; moreover, they are in consonance with Wang et al. [38] and Zhang et al. [40], two of the studies carried out on hepatic cell lines. In the first one, Li et al. [47] observed that OA attenuated adipose tissue IR through increasing gene expression of IRS-1, PI3K, and Akt, and by inhibiting the fructose-induced phosphorylation of IRS-1. These results suggest that OA could improve insulin signaling and glucose uptake through modulating the IRS-1/PI3K/Akt insulin pathway. In fact, previous studies have studied the role of PI3K/Akt in enhancing insulin signaling [5,64]. In the second one, OA improved the HOMA-IR and ADIPO-IR in adipose tissue insulin-resistant mice, probably by increasing effects on Akt phosphorylation and decreasing effects on JNK phosphorylation [48], since JNK activation has been shown to reduce insulin action through increasing phosphorylation of IRS-1 at serine [8,65]. Moreover, Li et al. [48] also showed that OA modified the polarization of macrophages in favor of M2 macrophages, which could also explain the improvement effects of OA in adipose tissue IR, since JNK phosphorylation has also been reported to promote polarization of adipose tissue macrophages (ATMs) to M1 macrophages [66], which are responsible for releasing proinflammatory cytokines in adipose tissue IR [67]. This idea coincides with the alleviating effects of OA on gene expression of iNOS, IL-6, TNF-α, IL-1β, and caspase 1, also reported by Li et al. [48]. A decrease in the phosphorylation of ERK in the adipose tissue of insulin-resistant mice was also reported by Li et al. [48], while in the study of Wang et al. [38], OA increased phosphorylation of ERK. This difference might be explained by the fact that OA was used in different kinds of experimental individuals. 

The eukaryotic enzyme AMPK’s main function is to regulate glucose uptake [68] through stimulating GLUT4 translocation [21,65,69], which is in consonance with the study conducted by Matumba et al. [50]. These authors reported a higher gene expression of both AMPK and GLUT4 in high-fructose-diet-fed neonatal rats treated with OA. AMPK has also been demonstrated to stimulate PI3K/Akt pathway [69], which could support the suppression of hepatic gluconeogenesis due to OA by increasing phosphorylation of AMPK, Akt, and PI3K in livers of type 2 diabetic mice [41]. This OA-induced phosphorylation of AMPK was also observed in rodent livers by Yunoki et al. [45] and by Wang et al. [42], demonstrating that OA lowers gene expression of TNF-α, IL-6, and IL-1β. The relief of inflammation might be explained by the OA-induced inactivation of NF-кB via dephosphorylation of IкB-α and of p65 [10,70], as was observed in livers of HFD-fed rats [46]. These results could indicate an improvement of insulin sensitivity and of glucose uptake, because NF-кB promotes the release of proinflammatory cytokines by suppressing the phosphorylation of AMPK [10]. 

Long-term inflammation leads to the generation of ROS in insulin target tissues [71], which supposes a greater activation of JNK and IKK [8], and consequently, higher levels of proinflammatory cytokines, further impairing insulin signaling [12]. Thus, combating OS is essential for alleviating IR, and in the present review, OA has exhibited antioxidant activities. Specifically, OA mitigated the production of ROS and GSSG, and also increased GSH in liver mitochondria of diabetic mice [42]. Moreover, OA augmented SOD [42,46], CAT [42,46], and GPx [46] activities, and decreased serum MDA [46] in livers of rodents with IR [42,46]. Since Wang et al. [42] reported an increment in the protein levels of Nrf2, these increased activities of antioxidant enzymes could be explained by the activation of OA of the transcription factor Nrf2, as was shown by previous authors [72,73]. Moreover, OA attenuated OS induced by Aroclor 1254 in the adipose tissue of insulin-resistant mice through downregulating the gene expression of NOX4, GCLC, and GCLM, decreasing serum MDA, and increasing serum SOD and CAT activities. OA also seems to mitigate the fructose-induced IR and OS, as was observed in the increasing effects of OA on GSH and CAT activities in the skeletal muscle of high-fructose-diet-fed neonatal rats [51]. 

All the aforementioned modulatory activity of OA on proinflammatory cytokines and on antioxidant enzymes was also shown by Gamede et al. [52] and Wang et al. [53]. In the first one, the application of OA mitigated in prediabetic rats the reduction of serum levels of SOD and GPx activities [52], suggesting that OA favors antioxidant activities. In the second one, OA also led to higher SOD and CAT activities, as well as a reduction in the serum levels of MDA and nitric oxide [53]. Moreover, Wang et al. [53] observed an enhancement in the insulin sensitivity in HFF-diet-fed rats treated with OA. Thus, OA could promote better sensitivity to insulin through reducing OS. 

This work has some strengths and limitations. The main strength of this research is that it provides a broad and overall picture of the effects of OA on the molecular mechanisms and signaling pathways involved in the development of IR and underlying OS in insulin-resistant animal models or cell lines. The large number of databases that have been used for the review and the utilization of the PRISMA 2020 protocol for the reporting of systematic reviews are also noteworthy [31]. One should note that the SYRCLE’s Risk of Bias (RoB) tool was also used for assessing the risk of bias of the animal studies. In addition, it should be noted that in order to reduce inter-examiner bias, the quality of the studies was evaluated by two independent reviewers. Moreover, a third reviewer resolved potential differences [74,75]. However, this review has several limitations. First, the selected articles are written in only English, possibly ignoring articles written in other languages; this is found to be a limitation. Second, the fact that we did not include grey literature in this systematic review would be another limitation of this study.

## 5. Conclusions

OA presents potential antioxidant, anti-inflammatory, and insulin-sensitizing properties of special interest for the treatment and prevention of IR. OA attenuates IR through improving insulin sensitivity and insulin signaling, which results in a better glucose homeostasis and inhibition of gluconeogenesis, as well as the mitigation of the OS that aggravates IR status by impairing insulin signaling pathways. Thus, the potential molecular targets of OA for alleviating IR are the modulation of the IRS-1/PI3K/Akt insulin signaling pathway, inactivation of FoxO1, and regulation of different MAPK pathways, which are implicated in the development of OS and of inflammation. However, more in vitro and in vivo studies should be addressed to obtain greater in-depth knowledge on the exact mechanism of interaction between OA and its target proteins, as well as the main source of ROS (activated NADPH oxidases, mitochondrial electron transport chain, and so on) targeted by OA. Moreover, the performance of randomized clinical trials is needed in order to elucidate its future use potential as a new and alternative therapeutic and preventive strategy for IR.

## Figures and Tables

**Table 1:** Specific filters applied on the search in each database.

Database	Search Field	Additional Filters
PubMed	All fields	Not applicable
Web of Science ^1^	Title, Abstract, and Keywords	Document types: articles, others, and clinical trial
Scopus	Title, Abstract, and Keywords	Document types: article
Cochrane	Title, Abstract, and Keywords	Document types: article
CINAHL ^2^	Full text	Not applicable

^1^ Search performed in “All databases”; ^2^ via EBSCOhost.

**Table 2:** Characteristics and results of the “in vitro” studies on the OA effects on insulin-resistant cell lines.

Author/Year	Subjects	Intervention and Groups	Main Findings
Wang X et al. [38] (2011)	QZG cells induced for IR with tBHP.	Intervention: Treatment with or without OA at 10 µM for 4 h, later exposition to tBHP, and finally stimulation with insulin. Groups: - CG: Non-insulin-resistant cells, non-treated with OA - Non-insulin-resistant cells only stimulated with insulin - Non-OA-treated insulin-resistant cells - OA-treated insulin-resistant cells	The exposition to tBHP decreased the insulin-stimulated phosphorylation of Akt and ERK, but it was inhibited by OA (*p* < 0.05). Non-significant (ns) actions on PGC-1α gene expression and on phosphorylation of p38 were observed.
Li M et al. [39] (2015)	HepG2 cells induced for IR with sodium oleate, except the CG.	Intervention: Insulin-resistant HepG2 cells treated with different doses of OA. Groups: - CG (non-insulin-resistant cells, non-treated with OA) - Non-treated insulin-resistant cells - Positive control → Rosiglitazone (RSG) - Experimental groups → OA-5 µM; OA-10 µM; OA-25 µM	↓ Content of glucose, IL-6, and TNF-α (*p* < 0.05) and ↓ protein expression of NF-кB (*p* < 0.01); ↑ IRS and GLUT4 protein expression (*p* < 0.01) in all OA groups vs. non-treated insulin-resistant cells.↓ ns of TNF-α levels at OA-5 µM vs. non-treated insulin-resistant cells.
Zhang Y et al. [40] (2020)	Insulin-resistant HepG2 cells induced with high concentrations of insulin.	Intervention and Groups: - CG: non-OA-treated insulin-resistant cells - OA group: OA-treated insulin-resistant cells - PGA-OA group: OA-loaded PGA-OA-treated insulin-resistant cells	↓ PTP1B protein expression and ↑ protein expression of Akt and IRS-1 in both insulin-resistant cell models treated with OA or PGA-OA. All changes *p* < 0.05.

QZG: human normal hepatocyte line; IR: insulin resistance; tBHP: tert-butyl hydroperoxide; CG: control group; Akt: protein kinase B; ERK: extracellular signal-regulated kinase; PGC-1α: PPARγ coactivator 1α; PPARγ: peroxisome proliferator-activated receptor γ; IL-6: interleukin-6; TNF-α: tumor necrosis factor-α; NF-кB: nuclear transcription factor kappa B; IRS: insulin receptor substrate; GLUT4: glucose transporter type 4; PGA: polygalacturonic acid; PTP1B: protein tyrosine phosphatase 1B.

**Table 3:** Characteristics and results of animal experimentation studies on the effect of oleanolic acid (OA) on signaling pathways impaired in insulin resistance and inflammatory/oxidative stress biomarkers in insulin-resistant animal models.

Author/Year	Subjects (n)	Intervention	Groups	Main Findings
Wang X et al. [41] (2015)	24 male C57BL/KsJ-Lepdb (db/db) mice	Intragastric administration of vehicle (0.5% CMC-Na), OA (250 mg/kg/day), or metformin (100 mg/kg/day) for 28 days.	Groups (*n* = 6 per group): - CG; - OA; - Metformin; - OA + metformin	↓ HOMA-IR; ↓ mRNA expression levels in liver (*p* < 0.001) of GP, PEPCK1, G-6-Pase, and GLUT2; ↓ ns of mRNA expression levels in liver of PGC-1α and ↑ ns of GS. ↑ AMPK, ACC, Akt, and PI3K and ↓ mTOR and CREB phosphorylation in livers; ↓ liver protein levels of PEPCK and of G-6-Pase. All changes reported are of OA group compared with CG.
Wang X et al. [42] (2013)	24 male C57BLKS/J lar-Lep^db/db^ mice, and 10 wild-type mice as control	Intraperitoneal injection of OA (20 mg/kg/day) for 14 days.	- CG - Untreated diabetic mice - OA-treated diabetic mice	↓ Liver protein expression of G-6-Pase and PEPCK; and ↑ PGC-1α gene expression and AMPK phosphorylation in livers.↑ Insulin-stimulated phosphorylation of Akt in livers. ↓ Mitochondrial ROS production and GSSG, and ↑ mitochondrial GSH in liver. ↑ Protein expression of Nrf2, GCLC, SOD, and CAT in liver. ↓ IL-6, IL-1β, and TNF-α both in serum and in liver. All changes (*p* < 0.05) in OA-treated diabetic mice compared with non-OA-treated diabetic mice.
Zeng X et al. [43] (2012)	Male C57BL/6J mice	Mice fed with a normal diet or an HFD during 10 weeks, and injection of STZ into HFD-fed rats to establish a T2DM model. Later, administration of 100 mg/kg/day of OA to T2DM mice for 2 weeks.	- CG (non-diabetic mice) - Untreated T2DM mice - OA-treated T2DM mice	phosphorylated-/total-Akt ratio similar in both diabetic mice groups; ↓ levels of p-Akt in non-treated T2DM mice vs. non-diabetic mice, while treatment with OA ↑ p-Akt levels in T2DM mice to levels of non-diabetic mice. In OA-treated T2DM mice, ↑ of phosphorylated-/total-FoxO1 ratio and ↓ in total FoxO1 protein (*p* < 0.05) compared to untreated T2DM mice and non-diabetic mice. All results measured in livers of mice.
Zhou X et al. [44] (2014)	Male C57BL/6J mice	Idem to Zeng et al. [43], but measurements of variables were undertaken after 4 weeks of OA administration (during OA treatment experiment), or after 2 weeks of OA administration followed by an OA-free HFD diet during 4 weeks (post-OA treatment).	- CG (non-diabetic mice) - Untreated T2DM mice - OA-treated T2DM mice	In liver of diabetic mice, the following was observed: ↓ total content of FoxO1, ↑ phosphorylation of FoxO1 and acetylation of FoxO1, and ↓ gene expression of G-6-Pase during and post OA treatment. ↑ Phosphorylation of AMPK-α and ACC; ↓ of the mature form of SERBP-1c in the livers of diabetic mice during OA treatment. All changes *p* < 0.05 vs. untreated diabetic mice.
Yunoki K et al. [45] (2008)	24 male Sprague Dawley rats	Administration of OA (50 mg/kg/day) or PEE (450 mg/kg/day) for 4 weeks	- CG: Normal fat diet - HFD group - HFD + OA - HFD + PEE	Downregulation of ACC, G-6-Pase, FoxO1, TNF-α, and IL-1β genes; and upregulation of genes of insulin receptor substrates and AMPK β-2 regulatory subunit in rat livers. All changes *p* < 0.01 vs. HFD-fed rats.
Xue C et al. [46] (2021)	30 Sprague Dawley Rats	Administration of OA (25, 50, and 100 mg/kg/day) for 8 weeks. Rats were simultaneously fed with an HFD or normal diet for 12 weeks.	Groups (*n* = 6 per group): - Normal-diet-fed rats - HFD-fed rats - HFD + OA 25 fed rats - HFD + OA 50 fed rats - HFD + OA 100 fed rats	↓ MDA and ↑ SOD, GPx, and CAT content; ↓ IL-1β, IL-6, and TNF-α overexpression; inhibition of the phosphorylation of IκB-α and p65 in liver tissues. All changes (*p* < 0.05), especially in rats treated with OA 50 or 100 vs. HFD-fed rats.
Li Y et al. [47] (2014)	24 male Sprague-Dawley rats	Fructose induced insulin-resistant rats and oral administration of 5 or 25 mg/kg/day of OA for 10 weeks.	Groups (*n* = 6 per group): - Control group: non-insulin-resistant rats. - Insulin-resistant rats: non-treated insulin-resistant rats; OA-5 mg; and OA 25 mg	↓ Adipo-IR; ↑ adipose mRNA expression of insulin receptor, IRS-1, PI3K, and Akt. ↑ IRS-1 protein expression, ↓ fructose-stimulated pIRS-1 protein expression, and of the ratio of pIRS-1 to total IRS-1 protein expression. ↑ Ratio of pAkt protein to Akt protein. All changes (*p* < 0.05) in the adipose tissue of insulin-resistant rats treated with OA 25 mg vs. non-treated insulin-resistant rats.
Li W et al. [48] (2021)	21 C57BL/6J male mice	All mice fed with HFD during 12 weeks. Later, administration with distilled water, or OA 25 mg/kg or OA 50 mg/kg per day by intragastric gavage for 4 weeks.	Groups (*n* = 7 per group): - CG: distilled water - OA-25: 25 mg/kg/day - OA-50: 50 mg/kg/day	↓ HOMA-IR and Adipo-IR index; ↑ phosphorylation of Akt and ↓ gene expression of iNOS, IL-6, TNF-α, IL-1β, and Caspase 1; ↓ macrophages M1 and ↑ macrophages M2 in eWAT of mice treated with OA-25 and -50. ↓ phosphorylation of ERK and JNK in eWAT of mice treated with OA-50. All changes (*p* < 0.05) vs. non-OA-treated HFD-fed mice.
Su S et al. [49] (2018)	40 male C57B6/J mice	Exposure to vehicle or Aroclor 1254 (100 µg/kg; PCBs) every 3 days for 10 weeks. Pretreatment with 50 mg/kg of OA for 1 h every 3 days for 10 weeks.	Groups (*n* = 10 per group): - CG - PCBs - PCBs + OA - PCBs + Vitamin C	↓ HOMA-IR, ↓ serum MDA, and ↑ serum SOD and CAT activity; ↓ mRNA expression of NOX4, GCLC, and GCLM in adipose tissue. All changes (*p* < 0.05) in favor of OA-pretreated mice vs. PCBs-treated mice.
Matumba MG et al. [50] (2019)	40 male Sprague Dawley rat pups	OA (60 mg/kg/day) administration by orogastric gavage during the second neonatal week. Duration of the experiment was 16 weeks.	- CG - OA - HF diet - HF diet + OA - HF diet + Met	↑ AMPK, GLUT4 and ↓ IL-6 and TNF-α gene expression (*p* < 0.001) in the skeletal muscle; and ↓ plasma concentration of IL-6 (*p* < 0.0001) and ns of TNF-α. All changes in OA-treated HF-fed rats vs. HF-fed rats.
Nyakudya T et al. [51] (2019)	30 Sprague Dawley rat pups	Administration of OA (60 mg/kg/day b.w.) by orogastric gavage in the second postnatal week. Duration of the experiments 14 days since their birth.	- CG (distilled water + DMSO) - OA - HF - OA + HF	The ↓ of GSH and CAT activity in the skeletal muscles of HF-fed rats was attenuated in OA-treated HF-fed rats (*p* < 0.05). Non-significant changes on GPx, SOD, and MDA.
Gamede M et al. [52] (2019)	36 male Sprague-Dawley rats	Treatment with OA or OA + dietary intervention for 12 weeks after previous administration of HFHC diet during 20 weeks to induce prediabetes.	Groups (*n* = 6 per group): Non-prediabetic control (NC); prediabetic control (PC); metformin (MET); MET + dietary intervention; OA; OA + diet	↓ Plasma levels of IL-6 and heart MDA concentration, and ↑ plasma level of SOD and GPx in OA-treated rats vs. prediabetic control group. All changes *p* < 0.05.
Wang S et al. [53] (2018)	36 male Sprague-Dawley rats	6 normal-diet-fed rats and 30 HFF-fed rats during the first four weeks. HFF-fed rats were intraperitoneally injected with tBHP during the last eight weeks of the experiments.	6 Groups (*n* = 6 per group): 6 HFF-fed rats were treated with OA and another 6 with nano-OA during the last 6 weeks of the experiments. 25 mg/kg/day	↓ Serum NO levels and ↑ serum CAT activity in OA and nano-OA groups; ↓ serum levels of MDA and ↑ serum SOD activity and ISI in nano-OA group. All changes (*p* < 0.05) vs. non-treated insulin-resistant rats.

CG: control group; OA: oleanolic acid; HOMA-IR: homeostatic model assessment for insulin resistance; GP: glycogen phosphorylase; PEPCK1: phosphoenolpyruvate carboxykinase 1; G-6-Pase: glucose-6-phosphatase; GLUT2: glucose transporter type 2; PGC-1α: PPARγ coactivator 1α; PPARγ: peroxisome proliferator-activated receptor γ; GS: glycogen synthase; AMPK: AMP-activated protein kinase; ACC: acetyl-CoA carboxylase; Akt: protein kinase B; PI3K: phosphatidylinositol-3-kinase; mTOR: mammalian target of rapamycin; CREB: cAMP-response element-binding protein; ROS: reactive oxygen species; GSSG: glutathione, oxidized form; GSH: glutathione; Nrf2: nuclear factor erythroid 2-related factor 2; GCLC: glutathione cysteine ligase catalytic subunit; SOD: superoxide dismutase; CAT: catalase; IL-6: interleukin-6; IL-1β: interleukin-1β; TNF-α: tumor necrosis factor-α; HFD: high-fat diet; STZ: streptozotocin; T2DM: type 2 diabetes mellitus; pAkt: phosphorylated-Akt protein at serine-473; FoxO1: forkhead box O1; SREBP-1c: sterol regulatory element-binding protein-1c; MDA: malondialdehyde; IRS: insulin receptor substrate; pIRS: phosphorylated-IRS at serine; eWAT: epidydimal white adipose tissue; JNK: c-Jun N-terminal kinase; ERK: extracellular signal-regulated kinase; NOX4: NADPH oxidase; GCLm, glutamate-cysteine ligase modifier subunit; RSG: rosiglitazone; HF: high-fructose; GPx: glutathione peroxidase; PEE: pomace ethanol extract; GLUT4: glucose transporter type 4; HFHC: high-fat high-carbohydrate; HFF: high-fat and fructose; tBHP: tert-butyl hydroperoxide; NO: nitric oxide; ISI: insulin sensitivity index.

## Data Availability

The data is contained within the article.
